# Effectiveness of Remote Patient Monitoring Equipped With an Early Warning System in Tertiary Care Hospital Wards: Retrospective Cohort Study

**DOI:** 10.2196/56463

**Published:** 2025-01-15

**Authors:** Pavithra Lakshman, Priyanka T Gopal, Sheen Khurdi

**Affiliations:** 1 Hospital Administration Ramaiah Memorial Hospital Bengaluru, Karnataka India

**Keywords:** continuous vitals monitoring, remote patient monitoring, early warning system, hospital wards, retrospective, cohort study, early deterioration monitoring, patient care, decision making, clinical information

## Abstract

**Background:**

Monitoring vital signs in hospitalized patients is crucial for evaluating their clinical condition. While early warning scores like the modified early warning score (MEWS) are typically calculated 3 to 4 times daily through spot checks, they might not promptly identify early deterioration. Leveraging technologies that provide continuous monitoring of vital signs, combined with an early warning system, has the potential to identify clinical deterioration sooner. This approach empowers health care providers to intervene promptly and effectively.

**Objective:**

This study aimed to assess the impact of a Remote Patient Monitoring System (RPMS) with an automated early warning system (R-EWS) on patient safety in noncritical care at a tertiary hospital. R-EWS performance was compared with a simulated Modified Early Warning System (S-MEWS) and a simulated threshold-based alert system (S-Threshold).

**Methods:**

Patient outcomes, including intensive care unit (ICU) transfers due to deterioration and discharges for nondeteriorating cases, were analyzed in Ramaiah Memorial Hospital’s general wards with RPMS. Sensitivity, specificity, chi-square test for alert frequency distribution equality, and the average time from the first alert to ICU transfer in the last 24 hours was determined. Alert and patient distribution by tiers and vitals in R-EWS groups were examined.

**Results:**

Analyzing 905 patients, including 38 with deteriorations, R-EWS, S-Threshold, and S-MEWS generated more alerts for deteriorating cases. R-EWS showed high sensitivity (97.37%) and low specificity (23.41%), S-Threshold had perfect sensitivity (100%) but low specificity (0.46%), and S-MEWS demonstrated moderate sensitivity (47.37%) and high specificity (81.31%). The average time from initial alert to clinical deterioration was at least 18 hours for RPMS and S-Threshold in deteriorating participants. R-EWS had increased alert frequency and a higher proportion of critical alerts for deteriorating cases.

**Conclusions:**

This study underscores R-EWS role in early deterioration detection, emphasizing timely interventions for improved patient outcomes. Continuous monitoring enhances patient safety and optimizes care quality.

## Introduction

Ensuring patient safety is vital in health care, whether in regular wards or critical care units. Swift detection of vital sign changes allows prompt adjustments in care levels. Delayed intervention for patient deterioration is linked to increased morbidity and mortality [1,2]. Hospitals use aggregate score based early warning systems (EWS) to detect vital sign deterioration. However, these rely on instantaneous measurements conducted hours apart [3], lack historical trends, and have sensitivity <80% for patient deterioration [4-6]. EWS based on continuous monitoring of the vital signs of hospitalized patients is expected to overcome the constraints of conventional, intermittent vital measurements. Multiple studies have shown that continuous vital signs monitoring is pivotal in identifying at-risk patients and facilitating timely interventions [7-14]. An automated system based on continuous monitoring of hospitalized patients’ vital signs in combination with EWS besides enhancing patient safety, has the potential to save health care practitioners (HCPs) time [11,15-17] mitigate errors [18], facilitate information sharing with relevant stakeholders for informed decision-making [19], and reduce costs [20].

To ensure patient safety, and enhance health care services, Ramaiah Memorial Hospital (RMH), has implemented an advanced commercial remote patient monitoring system (RPMS) in its wards. This system is designed for continuous monitoring of vital signs, featuring an EWS (R-EWS). Before the introduction of R-EWS, ward monitoring depended on manual spot checks every 4 hours to calculate the modified early warning score (MEWS), while continuous monitoring in intensive care units (ICUs) relied on a threshold-based alerting system. The implementation of the R-EWS significantly increased the time available for patient care by reducing the time nurses spent on routine monitoring, communication, and coordination. Majority of HCPs reported improvements in the level of care and overall patient safety, with many also noting an enhanced patient experience [21].

After several months of implementing R-EWS, the hospital sought to assess the system’s performance against previous monitoring practices. To this end, a retrospective analysis was conducted to evaluate its effectiveness in identifying patient deterioration. The performance of R-EWS was also benchmarked against simulated versions of MEWS (S-MEWS) and the ICU’s continuous threshold-based alert system (S-Threshold). The primary objective of this study was to determine how well the R-EWS, S-Threshold, and S-MEWS systems ensure patient safety by accurately distinguishing between deteriorating patients needing ICU transfer and stable patients ready for discharge.

## Methods

### Materials

The RPMS is a continuous, vital parameters monitoring system. It uses sensor sheets placed under the mattress that uses ballistocardiography technology to capture microvibrations from which heart rate (HR), respiratory rate (RR), and systolic blood pressure (SysBP) are computed in a noncontact manner [22-25]. Accessories like a pulse oximeter and temperature probe are used for contact-based measurements of oxygen saturation (SpO_2_) and temperature. The system incorporates a configurable and customizable multitiered EWS (R-EWS) for severity assessment, complemented by a user-friendly web dashboard and mobile app for centralized and remote ward monitoring. The hospital leverages this R-EWS for patient monitoring, reporting, and clinical decision-making.

### Study Design

This is a retrospective cohort study, conducted without blinding. It uses data from patients under R-EWS monitoring implemented in the general wards of RMH, a tier 1 city’s private hospital with over 500 beds. The study covers the period from December 1, 2022, to May 31, 2023. This study retrospectively analyzed the performance of the R-EWS in distinguishing between patients requiring ICU transfer and those stable enough for discharge. The study also retrospectively compared the R-EWS performance against 2 simulated frameworks: the S-MEWS and a S-Threshold. The vital signs data generated by the RPMS served as input for these simulated frameworks for the alert generation. Detailed descriptions of these frameworks are provided in the following subsections. To reduce bias all patients who satisfied the inclusion and exclusion criteria were used for analysis.

### Ethical Considerations

This study was approved by the RMH ethics committee (DRP/EFPl034/2023).

### R-EWS Framework

The R-EWS uses a dynamic 3-tiered alert (tier 1, tier 2, and tier 3) system to prioritize alerts based on the severity of vital signs deviations. This ensures that alerts are raised only when a patient’s condition deteriorates significantly. Each tier in the alert system is defined by specific threshold settings for monitored vital signs, as detailed in Table 1. The R-EWS focuses on observing trends over a set period rather than relying on isolated values. This observation window helps confirm sustained deviations in vital signs before triggering an alert. In addition, the tiers incorporate a cool-down period during which no alerts are generated, unless a higher tier is breached. This mechanism prevents frequent and redundant alerts. For tiers 1 and 2, the cool-down period is set at 3 hours. In contrast, tier 3 has no cool-down period and generates alerts every 10 minutes if the patient’s condition continues to meet the threshold criteria. This ensures that critical conditions are continuously monitored, allowing for immediate medical intervention when necessary.

**Table 1:** Vital signs thresholds for each alert tier in the remote patient monitoring early warning system framework, showing specific values for heart rate, respiratory rate, and blood pressure that trigger tier 1, tier 2, and tier 3 alerts, illustrating a stratified approach to patient monitoring.

Alert level			Tier 1 range		Tier 2 range		Tier 3 range
**HR^a^-contactless (beats per minute)**							
	High	120 < HR^a^ ≤ 129		130 < HR^a^ ≤ 139		HR^a^ ≥ 140	
	Low	40 >HR^a^ ≥ 36		—^b^		HR^a^ ≤ 35	
**RR^c^-contactless (breaths per minute)**							
	High	29 < RR^c^ ≤ 34		35 < RR^c^ ≤ 39		RR^c^ ≥ 40	
	Low	8 >RR^c^ ≥ 7		—^b^		RR^c^ ≤ 6	
**SysBP^d^-contactless (mm Hg)**							
	High	157 < SysBP^d^ ≤ 178		—^b^		SysBP^d^ ≥ 179	
	Low	102 >SysBP^d^ ≥ 82		—^b^		SysBP^d^ ≤ 81	
**SpO_2_^e^-contact accessory (%)**							
	Low	92 >SpO_2_^e^ ≥ 91		—^b^		SpO_2_^e^ ≤ 90	
**T^f^-contact accessory (°C)**							
	High	38.33 < T^f^ ≤ 38.88		—^b^		T^f^ > 38.89	
	Low	33 >T^f^ ≥ 33.88		—^b^		T^f^ < 33.87	

^a^HR: heart rate.

^b^Not applicable.

^c^RR: respiratory rate.

^d^SysBP: systolic blood pressure.

^e^SpO_2_: oxygen saturation.

^f^T: temperature.

### S-MEWS Framework

The S-MEWS simulations used vital sign values from the ranges defined in Table 2, excluding clinical signs such as urine output and mental status. To reduce bias, S-MEWS used the same vital signs scoring criteria as the MEWS previously used in the hospital, with scores calculated 4 times a day (12 AM, 6 AM, 12 PM, and 6 PM). The hospital-initiated notification and escalation protocols for a threshold of 4 or higher. Similarly, S-MEWS was calculated every 4 hours using a 1-hour median calculation for all vitals, triggering a positive alert for a score of 4 or higher. The vital signs data generated by the RPMS served as input for this simulated framework for alert generation.

**Table 2:** Vital signs threshold and the scoring system for simulated modified early warning system framework.

Parameters	3	2	1	0	1	2	3
Respiratory rate	—^a^	8≥	8< and ≤14	14< and <21	21≤ and <25	25≤ and <31	≤31
Heart rate	—^a^	40≥	40< and ≤60	60< and ≤100	100< and <111	111≤ and <130	≤131
Blood pressure	69≥	70≤ and ≤80	80< and ≤110	110< and <140	140≤ and <170	170≤ and <200	≤200
Oxygen saturation	91≥	91< and ≤93	93< and ≤95	95< to 100	—^a^	—^a^	—^a^
Temperature	—^a^	35≥	35< and ≤36	36< and ≤36.9	36.9< and ≤38	38< and ≤38.6	≤38.7

^a^—: not available.

### S-Threshold Framework

This EWS simulates traditional ICU monitors that provide continuous monitoring and trigger alarms whenever a specific vital sign exceeds its threshold. These single breach point thresholds can typically be muted for up to 2 minutes. The breach points for various vitals are as follows: High HR >120, Low HR <40, High RR >29, Low RR <8, High SysBP >157, Low SysBP <102, Low SpO_2_ <92, High *t*>38.33, and Low *t*<36. To ensure comparability with R-EWS, these threshold values correspond to the tier 1 breach points of R-EWS detailed in Table 1. Similar to S-MEWS, the vital signs data generated by the RPMS served as input for these simulated frameworks for alert generation.

### Study Participants

The study examined retrospective data from individuals who met the specific inclusion and exclusion criteria outlined as follows (Textbox 1).

**Eligibility criteria for study participants.**

**Inclusion criteria**

- All adult patients monitored on Remote Patient Monitoring System (RPMS) system from December 01, 2022, to May 31, 2023.
- Patients with complete intake and discharge information from hospital records.

**Exclusion criteria**

- All adult patients who were not monitored on the RPMS system.
- Patients aged <18 years.
- Patients whose data were less than 4 hours.

### Variables

The 2 study groups were deteriorating patients defined as requiring transfer to the ICU and nondeteriorating patients deemed stable for discharge from the hospital without ICU admission. The total, mean, and median number of alerts in the last 24 hours were calculated for each group for the 3 EWS frameworks. In addition, the average time from the first alert to deterioration was calculated for each framework within the final 24 hours. Specifically for the R-EWS framework, the proportions of patients receiving alerts and the number of alerts at various tiers and combination of vital alerts in both groups during the last 24 hours were computed.

Sensitivity and specificity values were calculated by classifying deteriorating participants with even one alert as true positives, deteriorating participants without alerts as false negatives, nondeteriorating participants without alerts as true negatives, and nondeteriorating participants with even one alert as false positives.

### Data Analysis

Descriptive statistics were used to provide the demographic alert counts of the study groups. It is expected that there may be some differences in demographics and significant difference in proportion of participants in each group as it represents the real patient population where only 4%-5% are expected to require advanced care.

A chi-square test assumed equal alert frequency distribution in both groups, with statistical significance indicated by *P*<.05. Using 6 degrees of freedom and a 1-tailed threshold of 12.592, the test assessed if the RPMS system effectively discriminates by generating more alerts in deteriorating patients than nondeteriorating ones.

## Results

From December 01, 2022, to May 31, 2023, 922 patients in general wards were monitored using RPMS. For this study, 905 patients met the predefined inclusion and exclusion criteria. Among them, 38 patients experienced deteriorations and were transferred to the ICU. The demographic information of the deteriorating and nondeteriorating group is given in Table 3. The age, gender ratio, and the average length of stay between the 2 groups are similar except the proportion of patients in each group. The median length of stay is 3 days.

Table 4 displays the descriptive alert statistics for the 2 groups across various frameworks. In addition, it illustrates the earliness of each framework in generating alerts for deteriorating group. Across different EWS the deteriorating group consistently showed higher average alerts per patient and median alerts compared with the nondeteriorating group. Specifically, in the R-EWS groups, the deteriorating group had 2.64 times higher alerts, S-Threshold had 2.69 times higher alerts, and S-MEWS had 3.4 times higher alerts. Continuous monitoring based on R-EWS and S-Threshold, produced more alerts for both deteriorating and nondeteriorating groups than the intermittent monitoring S-MEWS. The first alert to deterioration in continuous monitoring systems occurred at least 18 hours before ICU transfer, compared with 11 hours in intermittent monitoring.

**Table 3:** Demographic details of the participants included for analysis.

Parameters		Deteriorating	Nondeteriorating
Total number of patients, n (%)		38 (4)	867 (96)
Age (years), mean (SD) (range)		64.1 (14.28) (29-90)	54.81 (18.17) (18-98)
**Gender number, n (%)**			
	Male	22 (57.89)	527 (60.78)
	Female	16 (42.11)	340 (39.22)
Average length of stay (hours), mean (SD) (range)		95.6 (98.03) (5-448)	94 (112.36) (4-1817)
Total hours of monitoring, n		3632	81510

**Table 4:** Alert frequencies comparison between the frameworks.

Group		Alerts total	Alerts per patients, mean (SD)	Alerts, median (range)	Time of first alert to ICU^a^ Transfer (hours), mean (SD)
**R-EWS^b^**					
	D^c^	1402	36.89 (42.41)	17 (0-147)	18.7 (5.78)
	ND^d^	12100	13.96 (29.66)	3 (0-257)	—^e^
**S-Threshold^f^**					
	D	7031	185.03 (201.63)	102 (6-803)	20.1 (5.48)
	ND	59479	68.6 (98.64)	31 (0-940)	—
**S-MEWS^g^**					
	D	44	1.16 (1.6)	0 (0-5)	11.1 (8.53)
	ND	298	0.34 (0.88)	0 (0-6)	N/A^h^

^a^ICU: intensive care unit.

^b^R-EWS: remote patient monitoring early warning system.

^c^D: deteriorating.

^d^ND: nondeteriorating.

^e^Not available.

^f^S-Threshold: simulated threshold-based alert system.

^g^S-MEWS: simulated modified early warning system.

^h^N/A: not applicable.

**Figure 1:**
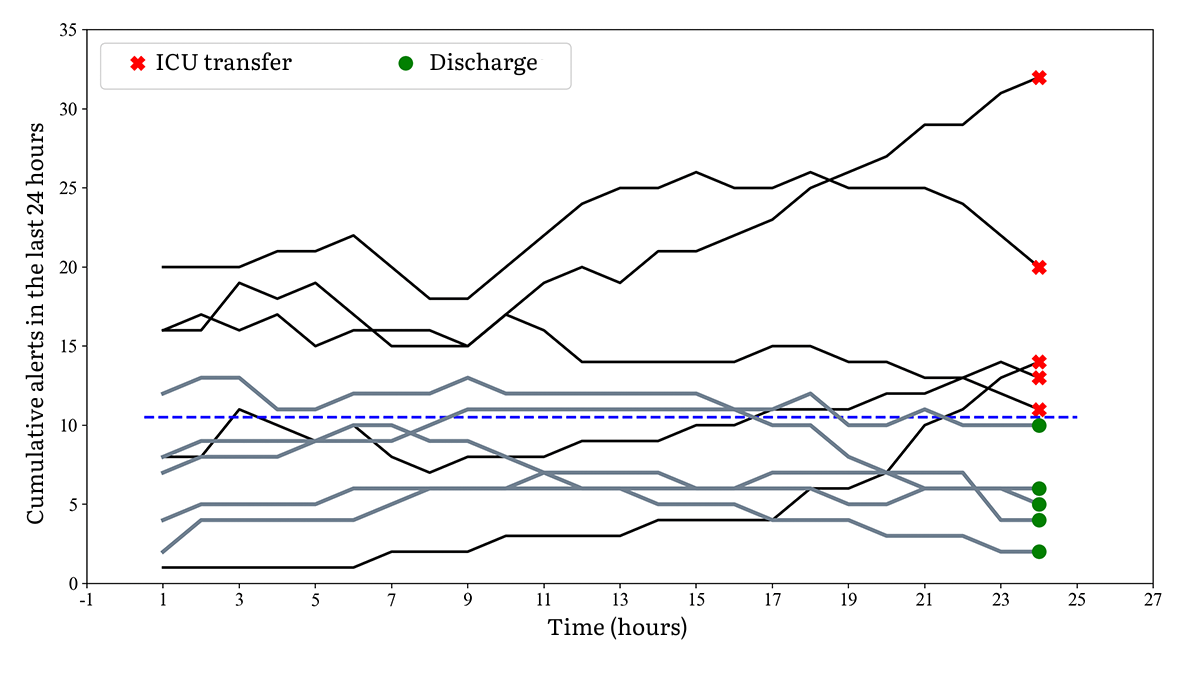
Representative 24-hour cumulative alerts generated in 5 participants each of those experiencing deterioration and nondeterioration in the R-EWS.

In Figure 1, for R-EWS, a depiction of 24-hour cumulative alerts is presented, illustrating the generated data from 5 participants in each category, those undergoing deterioration and those experiencing nondeterioration. The cumulative alerts were generated for the last 24 hours that lead to either a discharge or ICU transfer. These alerts were calculated every hour for the past 24 hours. The figure demonstrates the importance and discriminatory nature of the frequency of alert patterns in R-EWS for both groups. It clearly shows that more than 10 alerts in a day are primarily seen in patients whose conditions worsened and were subsequently transferred to the ICU, rather than in those who were discharged home.

The specificity and sensitivity of the different frameworks across the time points are given and the chi-square analysis is given in Table 5. R-EWS demonstrated high sensitivity (97.37%) but lower specificity (23.41%). S-Threshold achieved perfect sensitivity (100%) but had very low specificity (0.46%). In contrast, S-MEWS had moderate sensitivity (47.37%) and the highest specificity (81.31%), balancing fewer false positives with a lower detection rate. All frameworks showed statistically significant discriminative abilities (*P*<.001).

**Table 5:** Sensitivity and specificity and statistical test for discriminatory nature of different early warning systems frameworks and specificity.

Framework	Sensitivity (%) (TP^a^/TP^a^+FN^b^)	Specificity (%) (TN^c^/TN^c^ + FP^d^)	Chi-squared Value	*P* value
R-EWS^e^	97.37 (37/[37+1])	23.41 (203/[203+664])	27.71	<.001
S-Threshold^f^	100 (38/[38+0])	0.46 (4/[4+863])	44.7	<.001
S-MEWS^g^	47.37 (18/[18+20])	81.31 (705/[705+162])	39.2	<.001

^a^TP: true positive.

^b^FN: false negative.

^c^TN: true negative.

^d^FP: false positive.

^e^R-EWS: remote patient monitoring early warning system.

^f^S-Threshold: simulated threshold-based alert system.

^g^S-MEWS: simulated modified early warning system.

Table 6 presents data regarding the tier-wise alert numbers and the proportion of patients who received these alerts in both the deteriorating and nondeteriorating categories in the R-EWS framework. The deteriorating group received a significantly higher proportion of alerts across, especially in tier 2 and 3, compared with the normal discharge group. In tier 1, the deteriorating group had 1.36% high HR alerts, 3.35% high RR alerts, and 14.34% low SpO_2_ alerts, whereas the nondeteriorating group had 0.85% high HR alerts, 4.35% high RR alerts, and 26.05% low SpO_2_ alerts. In tiers 2 and 3, the deteriorating group shows consistently higher alert proportions, especially for critical parameters like SpO_2_. In tier 3, the deteriorating group received 73.61% low SpO_2_ alerts, compared with 54.84% in the nondeteriorating group. In addition, 26.32% of the deteriorating participants received high HR alerts in tier 1 versus 5.88% of the nondeteriorating participants, and 42.11% received high RR alerts versus 4.35%. Similarly, in tier 3, 76.32% of the deteriorating participants received low SpO_2_ alerts compared with 41.41% of nondeteriorating participants.

**Table 6:** Alert and patient distribution by tiers of the R-EWS^a^.

Study group				High HR^b^		Low HR		High RR^c^		Low RR		High SysBP^d^		Low SysBP		Low SpO_2_^e^
**Tier 1**																
	**Category: alerts/total (%)**															
		D^f^	19/1402 (1.36)		0/1402 (0)		47/1402 (3.35)		0/1402 (0)		11/1402 (0.78)		23/1402 (1.64)		201/1402 (14.34)	
		ND^g^	103/12100 (0.85)		0/12100 (0)		526/12100 (4.35)		18/12100 (0.15)		382/12100 (3.16)		562/12100 (4.64)		3152/12100 (26.05)	
	**Category: patient/total (%)**															
		D	10/38 (26.32)		0/38 (0)		16/38 (42.11)		0/38 (0)		3/38 (7.89)		8/38 (21.05)		14/38 (36.84)	
		ND	51/867 (5.88)		0/867 (0)		536/867 (4.35)		16/867 (1.85)		130/867 (14.99)		198/867 (22.84)		306/867 (35.29)	
**Tier 2**																
	**Category: alerts/total (%)**															
		D	12/1402 (0.86)		—^h^		10/1402 (1.36)		—		—		—		—	
		ND	32/12100 (0.26)		—		102/12100 (0.84)		—		—		—		—	
	**Category: patient/total (%)**															
		D	7/38 (18.42)		—		8/38 (21.05)		—		—		—		—	
		ND	22/867 (2.54)		—		60/867 (6.92)		—		—		—		—	
**Tier 3**																
	**Category: alerts/total (%)**															
		D	41/1402 (2.92)		0/1402 (0)		6/1402 (0.43)		0/1402 (0)		0/1402 (0)		0/1402 (0)		1032/1402 (73.61)	
		ND	297/12100 (2.45)		0/12100 (0)		73/12100 (0.60)		18/12100 (0.15)		189/12100 (1.56)		21/12100 (0.17)		6636/12100 (54.84)	
	**Category: patient/total (%)**															
		D	5/38 (13.6)		0/38 (0)		2/38 (5.26)		0/38 (0)		0/38 (0)		0/38 (0)		29/38 (76.32)	
		ND	13/867 (1.50)		0/867 (0)		19/867 (2.19)		3/867 (0.35)		17/867 (1.96)		6/867 (0.69)		359/867 (41.41)	

^a^R-EWS: remote patient monitoring early warning system.

^b^HR: heart rate.

^c^RR: respiratory rate.

^d^SysBP: systolic blood pressure.

^e^SpO_2_: oxygen saturation.

^f^D: deteriorating.

^g^ND: nondeteriorating.

^h^Not applicable.

Table 7 displays information on the vital alert numbers, both individually and in combination, along with the percentage of patients who received these alerts in both the deteriorating and nondeteriorating categories within the R-EWS group. Deteriorating group had a higher percentage of alerts across all vital signs compared with the nondeteriorating group. In total, 87.95% of alerts in the deteriorating group were low SpO_2_, compared with 80.89% in the nondeteriorating group In addition, combinations of alerts, such as HR and SpO_2_ and RR and SpO_2_, were significantly more prevalent in the deteriorating group. Notably, 26.32% of ICU transfer patients had HR and SpO_2_ alerts, whereas only 5.07% of normal discharge patients experienced these alerts. The presence of all 4 vital signs alerts (HR, RR, SysBP, and SpO_2_) is higher (21.05%) in the deteriorating group compared with only 2.31% in the nondeteriorating group. As a single parameter, HR generated the fewest alerts (504 alerts in total) but had the highest discriminatory nature, with alerts in escalated patients being 4.7 times more likely than in nonescalated patients.

**Table 7:** Alert and patient distribution by vitals for the R-EWS^a^ framework.

Parameter	Alerts/total alerts (%)		Patients/total patients (%)	
	D^b^	ND^c^	D	ND
HR^d^	72/1402 (5.14)	432/12100 (3.57)	13/38 (34.21)	63/867 (7.27)
RR^e^	63/1402 (4.49)	726/12100 (6.00)	16/38 (42.11)	248/867 (28.60)
SysBP^f^	34/1402 (2.43)	1154/12100 (9.54)	11/38 (28.95)	328/867 (37.83)
SpO_2_^g^	1233/1402 (87.95)	9788/12100 (80.89)	31/38 (81.58)	443/867 (51.10)
HR + RR	135/1402 (9.63)	1158/12100 (9.57)	6/38 (15.79)	38/867 (4.38)
HR + SysBP	106/1402 (7.56)	1586/12100 (13.11)	3/38 (7.89)	34/867 (3.92)
HR + SpO_2_	1305/1402 (93.08)	10220/12100 (84.46)	10/38 (26.32)	44/867 (5.07)
RR + SysBP	34/1402 (2.43)	1880/12100 (15.54)	5/38 (13.16)	109/867 (12.57)
RR + SpO_2_	1296/1402 (92.44)	10514/12100 (86.89)	12/38 (31.58)	144/867 (16.61)
SysBP + SpO_2_	1267/1402 (90.37)	10942/12100 (90.43)	8/38 (21.05)	180/867 (20.76)
HR + RR + SysBP	169/1402 (12.05)	2312/12100 (19.11)	2/38 (5.26)	26/867 (3.00)
HR + RR + SpO_2_	1368/1402 (97.57)	10946/12100 (90.46)	4/38 (10.53)	29/867 (3.34)
RR + SysBP + SpO_2_	1330/1402 (94.86)	11668/12100 (96.43)	3/38 (7.89)	70/867 (8.07)
HR + RR + SysBP + SpO_2_	1402/1402 (100.00)	12100/12100 (100.00)	8/38 (21.05)	20/867 (2.31)

^a^R-EWS: remote patient monitoring early warning system.

^b^D: deteriorating.

^c^ND: nondeteriorating.

^d^HR: heart rate.

^e^RR: respiratory rate.

^f^SysBP: systolic blood pressure.

^g^SpO_2_: oxygen saturation.

**Figure 2:**
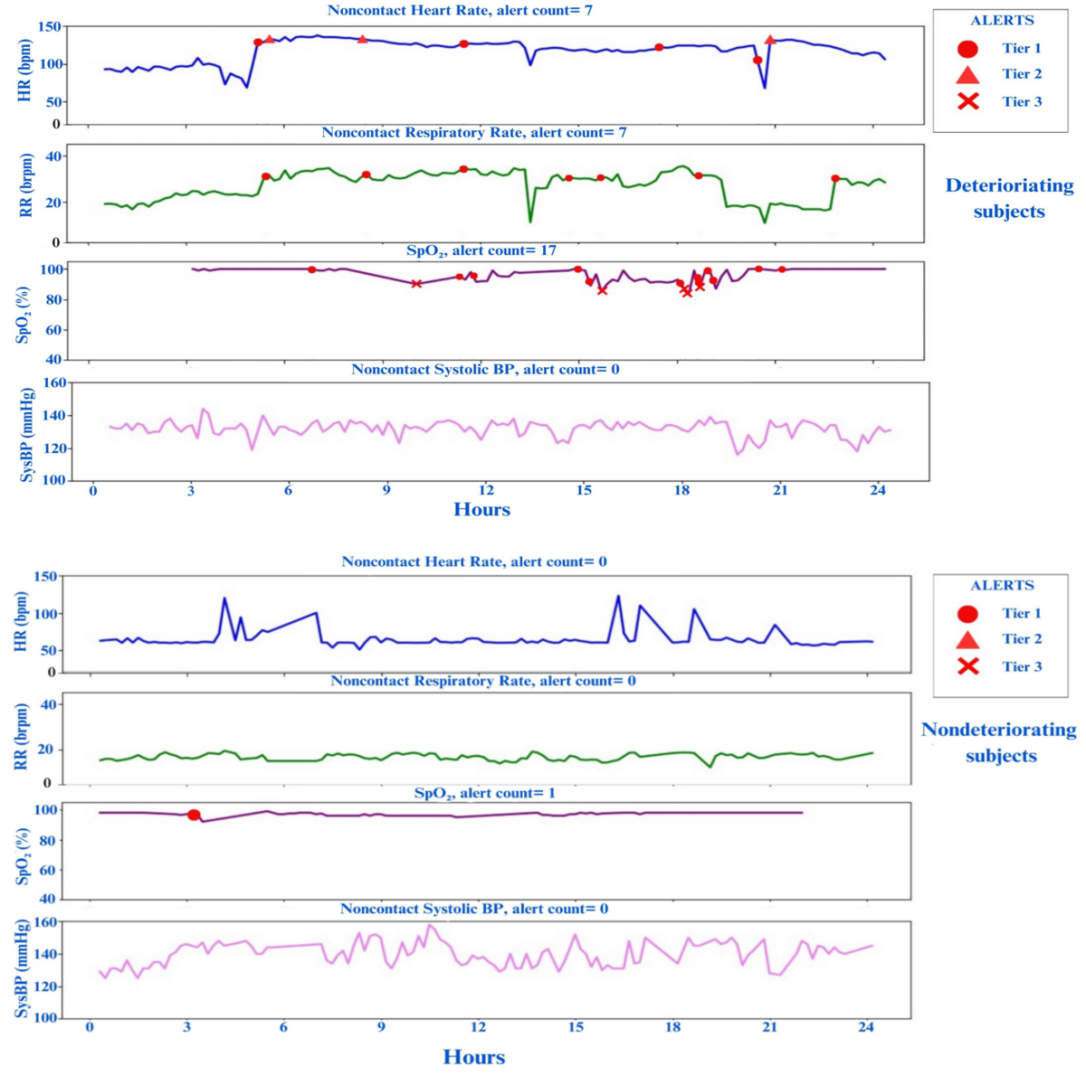
Representative 24-hour vital patterns depicting generated alerts in the remote patient monitoring early warning system framework (A) deteriorating and (B) nondeteriorating participants. BP: blood pressure; bpm: beats per minute; brpm: breaths per minute; SpO2: oxygen saturation.

Figure 2 illustrates trends in vitals and alerts generated by the R-EWS in the last 24 hours for both a deteriorating and a nondeteriorating participant. The deteriorating participant received numerous alerts before ICU transfer. Conversely, the nondeteriorating participant experienced minimal alerts.

## Discussion

### Principal Findings

This study aimed to evaluate the effectiveness of the R-EWS in ensuring patient safety by identifying deterioration in patients. Our findings reveal that R-EWS demonstrated a high sensitivity in identifying patients who were deteriorating and in need of ICU transfer. This was achieved with a relatively lower specificity from a single alert. In R-EWS, ICU transfer patients received a higher number of alerts, including alerts classified as critical, compared with their normal discharge counterparts.

As seen in Table 3, both groups had similar demographics though vastly different group sizes (38 deteriorating and 867 nondeteriorating). This is consistent with the expectation of ICU transfers or Death in wards to be 2%-6% [26]. Continuous monitoring identifies more patients developing complications than intermittent monitoring. This is supported by data in Table 5 where both R-EWS and S-Threshold, based on continuous monitoring, demonstrated a high sensitivity in alerting to potential patient deterioration. This is in stark contrast to the S-MEWS system, which, relying on intermittent hourly monitoring, showed a sensitivity of only 47%, a rate not clinically acceptable as it would miss deterioration in the majority of patients. These results align with existing literature indicating that continuous monitoring is more effective than intermittent and manual monitoring in early identification of complications and improving patient outcomes [27-29]. There is also established research showing that EWS based on 3- to 6-hour spot checks, a standard of care practice in most hospitals, can have sensitivity as low as 40% [4,5]. While the sensitivity of S-Threshold was 100% the specificity was nearly zero, indicating a high rate of false alerts for nondeteriorating patients. Alerting frequently on every patient is as ineffective as alerting on no patient, as these alarms are likely to be ignored and lead to alarm fatigue for health care workers. In this context, the R-EWS, with its balanced approach to minimal alerts with high sensitivity and moderate specificity emerges as a better choice.

The study highlights that continuous monitoring systems generate more alerts than intermittent systems as shown in Table 4 due to their higher frequency of measurements, a trend also observed in the scientific literature [29]. This study found that the intermittent monitoring-based S-MEWS generated 342 alerts which is 39 times less alerts than the 13,502 alerts generated in R-EWS and 194 times less than the 66,510 S-Threshold. By using a tier-based alerting with a cool down period R-EWS produced 5 times less alerts than S-Threshold. This reduction in alert number is important because alarm fatigue can occur when health care professionals become desensitized to the constant stream of alerts, potentially leading to slower response times or even missed alarms. This overload not only increases the workload but also pushes health care professionals toward burnout, a serious issue highlighted by Kristinsson et al [30] in 2022. The findings indicate that, on average, R-EWS generates 15 alerts per patient per 24 hours. This is a more manageable workload for health care professionals compared with the overwhelming 73 alerts per patient per 24 hours generated by the S-Threshold system. Considering it takes approximately 4 mins to assess an alert, addressing every alert would lead to spending 4.9 hours or over 50% of a standard shift on alerts which is impractical. In the case of R-EWS, an average of 15 alerts are generated per patient in 24 hours resulting in HCP spending about an hour collectively which is reasonable. In addition, this time is made up through the time saved from needing to manually check vitals and document and report the same for every patient.

The study also highlights that continuous monitoring systems provide alerts earlier than intermittent systems. As demonstrated in Table 4, one of the significant findings of our study is the ability of the R-EWS and S-Threshold systems to provide alerts an average of at least 18 hours before an ICU transfer. This represents a substantial improvement over the S-MEWS, which provides alerts an average of only 11 hours in advance. These results are consistent with what has been reported in the literature. For instance, continuous vital sign monitoring has been shown to warn of upcoming deterioration 8.3 hours in advance compared with 5.2 hours for periodic EWS [29]. Early detection of clinical deterioration enables timely and effective interventions, which not only improves patient outcomes but also has the potential to reduce associated health care costs [20,31].

The study highlights the effectiveness of the R-EWS system in distinguishing between patients at risk of deterioration and those with stable conditions. The discriminatory nature of the R-EWS is further evidenced by its trend of increased alerts in the deteriorating group compared with the nondeteriorating group seen in Table 4. This increased number of alerts in the deteriorating group aligns well with what is reported in the literature, where a higher frequency of alerts is typically associated with patients experiencing clinical deterioration [22].

R-EWS also demonstrated, as seen in Table 6, a higher proportion of patients receiving critical alerts (tier 2 and tier 3) among those experiencing deterioration compared with those without any escalations in care. Figure 2 shows the importance and discriminatory nature of the frequency of alert patterns in a random sampling from each group (deterioration and nondeterioration). In the last 24 hours health care providers can make more informed decisions based on whether a patient is improving or worsening. As shown in Figures 1 and 2, frequency of greater than 10 alerts in a day is primarily seen in patients whose conditions worsened and were transferred to the ICU, rather than in those who were discharged home. This can serve as a metric for daily evaluations and clinical decision support of discharge, observe further, or transfer or prepare for critical care.

Our findings as detailed in Table 7, highlight the critical importance of not only the volume but also the pattern and type of alerts. SpO_2_ alerts combined with HR or RR alerts are twice as likely in deteriorating patients compared with nondeteriorating ones, The integration of SpO_2_ alerts, especially in combination with HR or RR alerts, can provide a valuable early warning system for patient deterioration. SpO_2_ alerts combined with HR or RR alerts are 5 times and 2 times as likely in deteriorating patients compared with nondeteriorating ones, Furthermore, while all four vital alerts appeared in only 21% of deteriorating patients, they appeared in just 2% of nondeteriorating patients, underscoring this pattern as a critical marker. These findings suggest that monitoring systems should not rely solely on the volume of alerts but should also consider the context and combination of these alerts. Therefore, the development of alert algorithms that take into account the interdependence of various vital signs may enhance the predictive value of these systems.

While the study did not specifically focus on the cost-benefit aspect, it is worth noting the RPMS’s potential in reducing direct costs. Manual monitoring incurs high labor expenses and is prone to errors [32,33]. RPMS can lower these costs by automating monitoring and reducing HCP workload, particularly at this hospital with a 1:6 HCP-to-patient ratio. Early detection of patient deterioration, even if it prevents only 2%-5% of ICU admissions, can lead to substantial savings. ICU stays and emergency interventions are costly, with ICU costs (US $132.5 per day) far exceeding general ward costs (US $25 per day [34,35]. Despite the initial investment, RPMS’s cost-effectiveness in settings like this hospital is evident. It offers a viable solution for improving patient care and maintaining financial sustainability in Indian health care.

Mobilization is crucial for patient recovery. Traditional monitoring methods, which use wired sensors, restrict movement and are time consuming to set up, making mobilization difficult. The RPMS overcomes this by measuring 3 vital signs namely, HR, RR, and BP remotely, eliminating the need for tethering and allowing patients the freedom to move. During the study period, SpO_2_ and temperature were measured with wired sensors; these can be easily removed and reattached, minimizing disruption. Thus, RPMS significantly enhances patient mobility while ensuring continuous monitoring. RPMS is now equipped with wireless sensors for these measurements, which further enhances mobility without limitations. The contactless measurement of the 3 vital signs saves nursing time by eliminating the need to detach and reattach sensors each time the patient leaves and returns to bed. There is still a limitation in the current system because it only measures vitals when the patient is on the bed and not during ambulatory activities.

There are practical challenges associated with continuous monitoring for every patient that need consideration; high initial setup costs, the necessity for comprehensive staff training, and potential resistance to new technology to name a few. Integration with existing hospital systems can be complex, requiring technical expertise to ensure reliability and accuracy. Patient comfort and compliance are also crucial. Alarm fatigue from frequent alerts is another concern along with data privacy and security [36,37]. There is also a risk of overreliance on automation, which could lead to neglect of thorough patient assessments, deskilling which can lead to missing subtle signs of deterioration [38]. Addressing these challenges and fine-tuning RPMS in the future is necessary for the successful adoption and sustainability of RPMS in health care settings.

### Limitations

The limitations of the study include the fact that the study wards had a diverse patient demographic. Such diversity, while reflective of a real-world scenario, could introduce variables that affect the generalizability of the findings. The comparison between the frameworks is unequal due to the inherent nature of each framework, which results in different sets of observations. The simulation of the MEWS only used vital signs, due to the unavailability of data on urine output and consciousness levels. The inclusion of these parameters could potentially have enhanced S-MEWS’s sensitivity, though it does speak to the need for a more labor-intensive approach for MEWS compliance.

### Conclusions

The study underscores the transformative impact of the RPMS using continuous monitoring and R-EWS alerting system on patient safety, emphasizing early deterioration. Despite certain limitations, the RPMS proves to be effective for improving patient safety, advocating for broader implementation. To further validate these findings, controlled trials are essential. In addition, more detailed costing data are crucial for a comprehensive cost of care analysis and to better understand the system’s overall efficiency. Future studies should also involve collaboration with machine learning and artificial intelligence experts to optimize the system’s specificity and sensitivity.

## Abbreviations

EWS: early warning systems
HCP: health care provider
HR: heart rate
ICU: intensive care unit
MEWS: modified early warning score
R-EWS: Remote patient monitoring early warning system
RMH: Ramaiah memorial hospital
RPMS: remote patient monitoring system
RR: respiratory rate
S-MEWS: simulated modified early warning system
S-Threshold: simulated threshold-based alert system
SpO2: oxygen saturation
SysBP: systolic blood pressure
